# Draft Genome Sequences of Two *Pseudoalteromonas* Strains Isolated from Roots and Leaf Blades of the Seagrass *Zostera marina*

**DOI:** 10.1128/genomeA.00010-16

**Published:** 2016-02-18

**Authors:** Alexandra Alexiev, Megan L. Krusor, Guillaume Jospin, Jenna M. Lang, Jonathan A. Eisen, David A. Coil

**Affiliations:** a UC Davis Genome Center, University of California, Davis, Davis, California, USA; b Department of Earth and Planetary Sciences, University of California, Davis, Davis, California, USA; c Department of Evolution and Ecology, University of California, Davis, Davis, California, USA; d Department of Medical Microbiology and Immunology, University of California, Davis, Davis, California, USA

## Abstract

Here, we present the draft genome sequences for *Pseudoalteromonas* sp. strain UCD-33C and *Pseudoalteromonas lipolytica* UCD-48B. *Pseudoalteromonas* sp. UCD-33C was isolated from *Zostera marina* roots and *P. lipolytica* UCD-48B from *Z. marina* leaf blades, both collected in Woods Hole, MA. These assemblies contain 4,479,285 bp and 4,592,435 bp, respectively.

## GENOME ANNOUNCEMENT

*Pseudoalteromonas lipolytica* was first isolated from Yangtze River estuary seawater. It is Gram-negative, motile, and strictly aerobic, has rod-shaped cells, and produces exopolysaccharides (1). Some *Pseudoalteromonas* strains exhibit antimicrobial abilities that are inhibitory to cystic fibrosis-associated opportunistic pathogens (2). *Pseudoalteromonas* sp. strain UCD-33C and *P. lipolytica* UCD-48B were both isolated from seagrass (*Zostera marina*) collected in Woods Hole, MA. *Pseudoalteromonas* sp. UCD-33C came from roots, whereas strain UCD-48B was isolated from leaf blades. This culturing project was done as part of a collaboration between researchers at the University of California, Davis, CA, and University of Oregon, Eugene, OR, called The Seagrass Microbiome Project (http://www.seagrassmicrobiome.org). The project seeks to characterize and analyze the microbial communities living in and on seagrasses.

Bacterial isolates were grown and double-dilution struck on Luria broth (LB) agar (Difco), seawater agar (SWA), 10% diluted seawater agar (SW10), and *Azotobacter* isolation medium agar (NFM). The isolates were incubated at 25°C for 1 to 21 days. Scrapings were then frozen in 25% glycerol for long-term storage. The isolates were later thawed and grown in seawater nutrient agar medium (ATCC medium 2205, using Instant Ocean instead of synthetic seawater). DNA was subsequently extracted from a fresh overnight culture using the Wizard genomic DNA purification kit (Promega).

A paired-end library was produced using a Nextera DNA sample prep kit (Illumina) and sequenced on an Illumina HiSeq (250 bp paired-end reads). Sequencing of *Pseudoalteromonas* sp. UCD-33C resulted in 807,945 reads and approximately 90× coverage. The genome size was 4,479,285 bp, and the G+C content was 41.3%. Sequencing of *P. lipolytica* UCD-48B yielded 885,488 reads and approximately 96× coverage. Its genome size was 4,592,435 bp and had 41.4% G+C content. The sequences were processed using the A5-miseq assembly pipeline (3, 4), which automates error correction, data cleaning, contig assembly, scaffolding, and quality control. The completeness of the genome was assessed using PhyloSift (5), which utilizes a list of 37 highly conserved single-copy marker genes (6). One copy of each marker gene was found in the sequences. Automated annotation was done using the RAST annotation server (7). A combination of BLAST and phylogenetic trees using the full-length assembled 16S rRNA sequences revealed strain UCD-48B to belong to *P. lipolytica*. However, the placement of the UCD-33C strain was ambiguous, falling into a polyphyletic and poorly resolved group, making it impossible to determine a species without further work.

### Nucleotide sequence accession numbers.

The genome sequence for *Pseudoalteromonas* sp. UCD-33C has been deposited at DDBJ/EMBL/GenBank under the accession no. LJTB00000000. The version described in this paper is no. LJTB00000000.1. The genome sequence for *P. lipolytica* UCD-48B has been deposited at DDBJ/EMBL/GenBank under the accession no. LJTC00000000. The version described in this paper is no. LJTC00000000.1.
